# The postoperative prognosis of older intertrochanteric fracture patients as evaluated by the Chang reduction quality criteria

**DOI:** 10.1186/s12877-022-03641-z

**Published:** 2022-12-01

**Authors:** Miao He, Jian Liu, Xu Deng, Xiaoxing Zhang

**Affiliations:** Department of Orthopaedic Surgery, Chongqing Emergency Medical Center (Chongqing University Central Hospital), No. 1 Jiankang Road, Chongqing, 400010 China

**Keywords:** Chang reduction quality criteria, Older adults, Intertrochanteric fractures, Mortality

## Abstract

**Objective:**

The aim of this study was to investigate the relationship between the Chang reduction quality criteria (CRQC) and the outcome of intertrochanteric fractures in older adults according to follow-up time.

**Methods:**

This was a retrospective analysis of 389 older adult patients with intertrochanteric fractures treated surgically from January 2019 to June 2021, including 130 males and 259 females aged 84.6 (77.5–89.7) years. Patient survival was determined by telephone as the time between admission to hospital for fracture and death or until the study deadline (June 1, 2022). According to the CRQC, the patients were divided into the Poor, Acceptable, and Excellent groups. Univariate and multivariate Cox proportional hazard models were used to assess the association between CRQC and all-cause mortality in older adult intertrochanteric fractures at 1 year and the total follow-up time. Further subgroup analysis was performed according to different clinical and biological characteristics to improve the accuracy of the results.

**Results:**

The mortality rates were 24.7% and 15.4% at 1 year and the total follow-up time, respectively. Both at one year and the total follow-up time, the mortality of the CRQC-Excellent group was significantly lower than that of the CRQC-Acceptable group (p.adj < 0.05) and the CRQC-Poor group (p.adj < 0.05). After multifactor adjustment, CRQC grades of Acceptable and Poor were independent risk factors affecting the overall and 1-year mortality. In addition, advanced age, ≥ 1 comorbidities, ASA 3 + 4, and prolonged preoperative waiting time were independent risk factors for survival at the total follow-up time. At 1 year, only ASA 3 + 4 and prolonged preoperative waiting time were independent risk factors for survival. Subgroup analysis according to different characteristics at the total follow-up time and at one year showed that in most subgroups, a decrease in the CRQC grade was significantly associated with an increase in all-cause mortality (p for trend < 0.05).

**Conclusions:**

This study highlights that CRQC grades of Acceptable and Poor are associated with increased all-cause mortality in older adult intertrochanteric fractures. We should attempt to achieve good reduction of these fractures.

## Background

Intertrochanteric fractures of the femur are more likely to occur in older individuals with osteoporosis; most of these fractures are unstable and associated with high mortality and disability rates. Clinical treatment of these fractures is difficult to some extent. With the aging of the population, the incidence of intertrochanteric fractures has been increasing annually [1–3]. At present, early fixation surgery is the mainstream treatment, allowing patients to engage in early functional exercise and outdoor activities, thus reducing the risk of complications and improving quality of life; commonly used implants are mainly intramedullary and extramedullary fixation [4]. Intramedullary fixation provides good biomechanical stability and is currently the gold standard for the treatment of intertrochanteric fractures. Intramedullary fixation is commonly performed with PFNA and INTERTAN fixation systems [5, 6].

The Guidelines of The National Institute for Health and Care Excellence (NICE) emphasize that getting patients out of bed early after surgery can reduce mortality [7]. To achieve these goals, the fracture needs to be fixed in a stable structure that will fully support load-bearing, thereby reducing pain and improving function [8]. Poor stability after fracture fixation can lead to limb shortening, hip pain, dysfunction, and even repeat surgery [9, 10], resulting in a delay in getting the patient out of bed. Reduction quality is one factor determining stability after fracture fixation [11, 12].

**Fig. 1:**
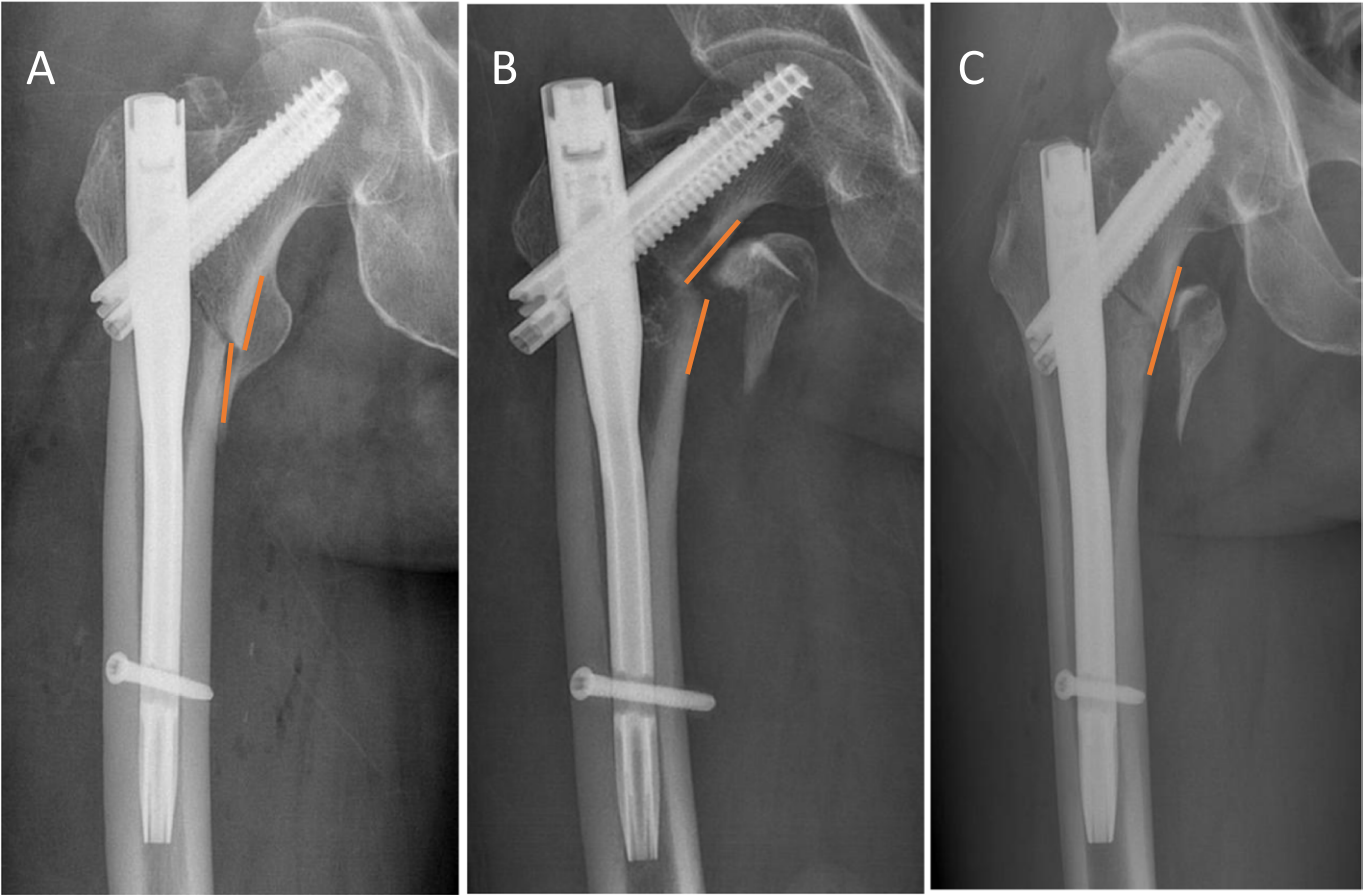
Postoperative X-ray of intertrochanteric fractures. **A** X-ray shows positive medial cortical support, meaning that the inferior edge of the medial cortex of the femoral head-neck fragment is medial to the superior edge of the medial cortex of the femoral shaft with a displacement of less than one cortical thickness. **B** X-ray shows negative medial cortical support, meaning that the inferior edge of the medial cortex of the femoral head-neck fragment is lateral to the superior edge of the medial cortex of the femoral shaft, regardless of displacement distance. **C** X-ray shows neutral medial cortical support, meaning that the medial cortex of the head–neck fragment and the femoral shaft are in smooth contact

There are different criteria for evaluating the quality of intertrochanteric fracture reductions, the most widely used of which are the criteria developed by Baumgaertner et al. (Baumgaertner reduction quality criteria, BRQC) [13, 14]. Later, Chang et al., based on positive medial cortical support (PMCS; Fig. 1) and negative medial cortical support (NMCS; Fig. 1), proposed a new reduction quality standard named the Chang reduction quality criteria (CRQC) [15] (Table 1). Studies have shown that the CRQC is more reliable than the BRQC in assessing stability after fracture fixation [16].

**Table 1:** Chang reduction quality criteria (CRQC)

Item	Score
I. Alignment	
a. Anteroposterior view: normal or slightly valgus neck-shaft angle^a^	1
b. Lateral view: less than 20° of angulation	1
II. Displacement	
a. Anteroposterior view: neutral or positive medial cortical support^b^	1
b. Lateral view: smooth anterior cortical contact^c^	1
Reduction quality	
Excellent	4
Acceptable	2 or 3
Poor	0 or 1

^a^Slightly valgus means a valgus angle of no more than 10°[15]

^b^Neutral medial cortical support is shown in Fig. 1

^c^Displacement of less than half of the cortex thickness

At present, in many studies, researchers have investigated the factors influencing mortality after intertrochanteric fractures in older patients. Advanced age [17], male sex [18], presence of comorbid diseases [19], high American society of anesthesiologists (ASA) score [20], low hemoglobin level [21] and blood transfusion [22] have been described as the predictive factors for mortality in patients with intertrochanteric fractures. Furthermore, the impact of preoperative waiting time and type of anesthesia on mortality risk remains controversial [23, 24]. To the best of our knowledge, there are no studies published on the efficacy of the CRQC in evaluating the quality of femoral intertrochanteric fracture reduction nor the impact of reduction quality on mortality.

The main objective of this article is to assess the impact of reduction quality in regard to the CRQC on patient survival after the intramedullary nail fixation of intertrochanteric femoral fractures in older patients.

## Materials and methods

### Study design and patients

A total of 389 patients with intertrochanteric fractures were admitted to the hospital between January 2019 and June 2021 for this study. All patients were fixed with INTERTAN or PFNA systems. The doctors in charge of the operation were senior surgeons. The choice of internal fixation was based on surgeon preference and experience. On the first day after surgery, if the conditions permitted, we encouraged all patients to perform partial weight bearing activities, regardless of the quality of their reduction. The inclusion criteria were as follows: (1) diagnosis of intertrochanteric fracture (AO 31A1 or 31A2) [25]. (2) Age ≥ 65 years. (3) Low-energy fractures, fresh fractures not older than 3 weeks. (4) Complete medical history. The exclusion criteria were as follows: (1) Age < 65 years. (2) High-energy fractures, old fractures, and pathological fractures. (3) Prior conservative treatment. (4) Refusal of or loss to follow-up.

### Data collection

We retrospectively recorded age, sex, fracture type, fracture orientation, number of comorbidities, American Society of Anesthesiologists (ASA) classification, type of anesthesia, preoperative waiting time, blood transfusion, preoperative hemoglobin, and CRQC grade for the patients. The comorbidity data of these patients were identified using the codes of the 10th Revision of the International Classification of Diseases.

The main exposure variable was the reduction quality as assessed by the CRQC. The CRQC was assessed by two independent assessors (LJ and DX) blinded to each other's scores. Cases of disagreement were resolved with the assistance of a third assessor (HM).

### Follow-up and study endpoint

The patient was specifically contacted and followed up by telephone by an experienced clinical nurse who was not aware of the patient's fracture reduction. Patients were followed up by telephone once a month for the first three months after discharge, then every three months until one year, and every six months thereafter. Follow-up time was defined as the time between hospitalization and the date of death or the last follow-up (June 30, 2022).

### Statistical analysis

IBM SPSS 26.0 (IBM Corp. New York, USA) were used for statistical analyses. The significance level was set to 0.05. Continuous variables are expressed as the mean ± standard deviation (SD) or median (interquartile range, IQR). The Shapiro‒Wilk test was used to assess the normality of the distribution of variables. The Kruskal‒Wallis H test was used for comparative analysis between the 3 groups of data that did not conform to a normal distribution. One-way ANOVA was used for comparative analysis between the 3 groups for normally distributed data. Categorical variables are expressed as frequencies (percentages) and were compared by the χ2 test. Hazard ratios (HR) and 95% confidence intervals (CIs) for the risk of death were calculated by one-way Cox regression analysis, incorporating the influencing factors (*p* < 0.10) into a multifactorial Cox proportional risk model. We also performed subgroup analyses for age, sex, fracture subtype, fracture orientation, number of comorbidities, ASA score, blood transfusion, and hemoglobin level. For the subgroup analyses, we did not perform multivariate adjustment because of the very small number of events.

### Ethics approval

This retrospective study involving human participants was conducted in accordance with the ethical standards of the institutional and national research committee and with the 1964 Helsinki Declaration and its later amendments or comparable ethical standards. The study was approved by the Ethics Committee of the Central Hospital affiliated with Chongqing University. Informed consent was obtained from all participants.

## Results

### Patient characteristics

A total of 389 patients with intertrochanteric fractures were included in this study, and their characteristics are summarized in Table 2. The average age of these participants was 84.6 (IQR, 77.5–89.7) years, with females accounting for the majority of patients (66.6%). All-cause mortality occurred in 96 patients over the total follow-up period (mortality rate: 24.7%). At the 1-year follow-up, death from any cause had occurred in 60 patients (mortality: 15.4%).

**Table 2:** Patient characteristics according to CRQC grade

Variable	Total (*n* = 389)	CRQC grade			*P* value
		Poor (*n* = 26)	Acceptable (*n* = 141)	Excellent (*n* = 222)	
Age (years)	84.6 (77.5–89.7)	85 (71.8–89.2)	84 (77.8–89.7)	84.6 (77.9–89.7)	0.745^a^
Female, n (%)	259 (66.6)	18 (69.2)	90 (63.8)	151 (68.0)	0.681^b^
31A2, n (%)	310 (79.7)	24 (92.3)	116 (82.3)	170 (76.6)	0.107^b^
Right-side fracture, n (%)	246 (63.2)	16 (61.5)	79 (56.0)	151 (68.0)	0.068^b^
Comorbidities, n (%)					** < 0.001**^**b**^
None	85 (21.9)	2 (7.7)	22 (15.6)	61 (27.5)	
1	139 (35.7)	12 (46.2)	40 (28.4)	87 (39.2)	
2 or more	165 (42.4)	12 (46.2)	79 (56.0)	74 (33.3)	
ASA score, n (%)					0.127^b^
1 + 2	43 (11.1)	6 (23.1)	13 (9.2)	24 (10.8)	
3 + 4	346 (89.0)	20 (76.9)	128 (90.8)	198 (89.2)	
Type of anesthesia, n (%)					**0.017**^**b**^
General	145 (37.3)	10 (38.5)	60 (42.6)	75 (33.8)	
Combined spinal and epidural + spinal	232 (59.6)	14 (53.9)	81 (57.5)	137 (61.7)	
Nerve block	12 (3.1)	2 (7.7)	0 (0.0)	10 (4.5)	
Preoperative waiting time (days)	3 (2–5)	4 (2–6.25)	3 (2–6)	3 (2–4)	0.097^a^
Hemoglobin (g/l)	107 (93–119)	92.5 (77–112.8)	104 (93.5–116.5)	108.5 (94.8–120)	**0.017**^**a**^
Blood transfusion, n (%)	219 (56.3)	16 (61.5)	93 (66.0)	110 (49.6)	**0.008**^**b**^
1-year death, n (%)	60 (15.4)	14 (53.9)	32 (22.7)	14 (6.3)	** < 0.001**^**b**^
Total death, n (%)	96 (24.7)	14 (53.9)	45 (31.9)	37 (16.7)	** < 0.001**^**b**^

*ASA* American Society of Anesthesiologists, *CRQC* Chang reduction quality criteria

^a^Kruskal‒Wallis H test

^b^χ2 test

**Fig. 2:**
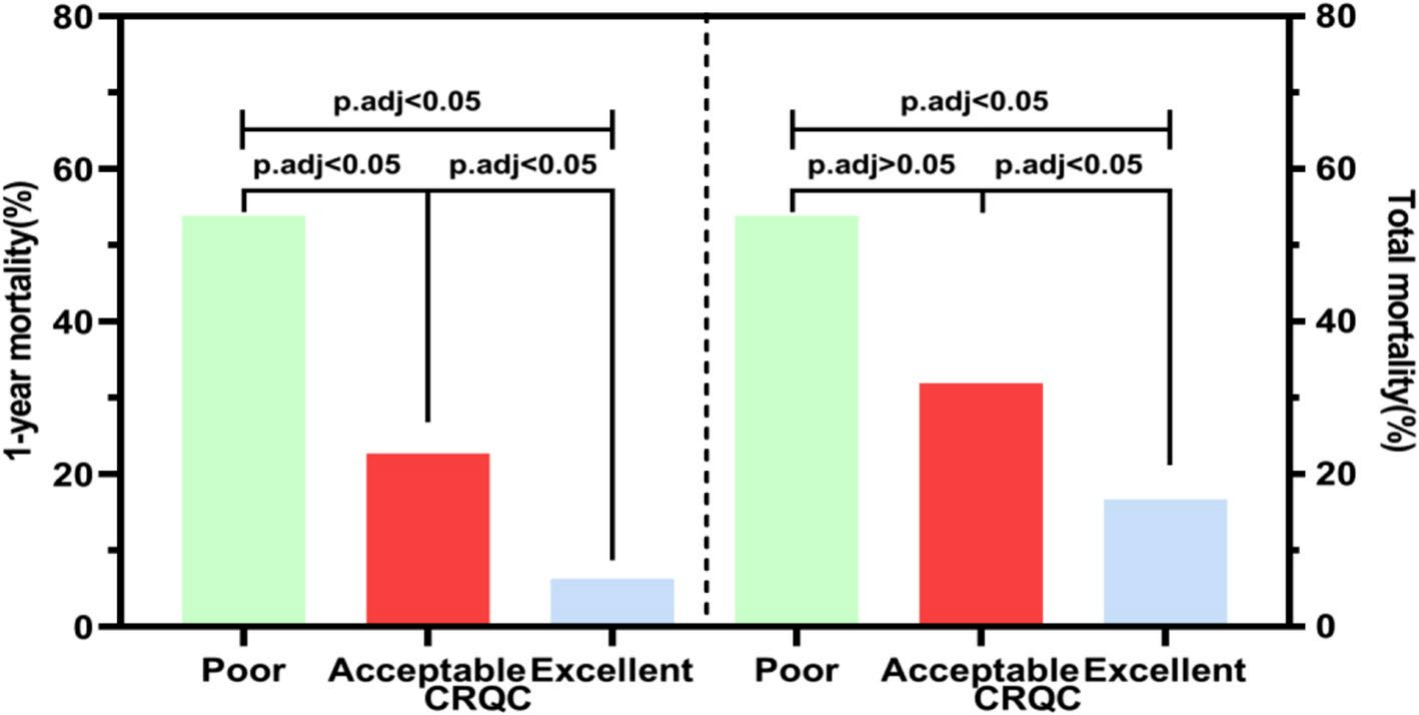
Column diagram comparing mortality in older adult intertrochanteric fracture patients at the 1-year and total follow-up for different Chang reduction quality criteria (CRQC) grades. *P* values obtained using the chi-square test

As shown in Table 2, there were no statistically significant differences in the comparison of sex, age, fracture subtype, orientation of the fracture, ASA score or preoperative waiting time among the 3 CRQC groups (Poor, Acceptable, Excellent), whereas statistically significant differences were found in the comparison of the number of comorbidities, type of anesthesia, blood transfusion and hemoglobin level (P < 0.05). As shown in Table 2 and Fig. 2, at the total follow-up, the morbidity of the CRQC-Excellent group was significantly lower than that of the CRQC-Acceptable (16.7% vs. 31.9%, p.adj < 0.05) and CRQC-Poor groups (16.7% vs. 53.9%, p.adj < 0.05). The mortality of the CRQC-Acceptable group was lower than that of the CRQC-Poor group, but the difference was not significant (31.9% vs. 53.9%, p.adj > 0.05). At the one-year follow-up, the CRQC-Excellent group had significantly lower mortality than the CRQC-Acceptable (6.3% vs. 22.7%, p.adj < 0.05) and CRQC-Poor groups (6.3% vs. 53.9%, p.adj < 0.05), and mortality in the CRQC-Acceptable group was significantly lower than that in the CRQC-Poor group (22.7% vs. 53.9%, p.adj < 0.05) (Fig. 2).

### Clinical variables predicting mortality

The relationship between clinical variables and the prognosis of intertrochanteric fractures in older patients is listed in Tables 3 and 4. At the total follow-up, according to univariate Cox analysis, increasing age, fracture type (31A2), ≥ 1 comorbidity, ASA scores 3 + 4, longer preoperative waiting time, lower hemoglobin level, blood transfusion and lower CRQC grade were significantly associated with increased overall mortality (*p* < 0.10). At the 1-year follow-up, older age, fracture type (31A2), ASA scores 3 + 4, longer preoperative waiting time, decreased hemoglobin levels, blood transfusions and lower CRQC grade were found to be significantly associated with increased overall mortality by univariate Cox analysis (*p* < 0.10).

**Table 3:** Univariate and multivariate Cox regression analyses of factors associated with total follow-up all-cause mortality

Variable	Univariate		Multivariate	
	HR (95% CI)	*P* value	HR (95% CI)	*P* value
Age (per 1 year increase)	1.1 (1.0–1.1)	** < 0.001**	1.0 (1.0–1.1)	**0.006**
Sex (Female vs. Male)	1.3 (0.8–1.9)	0.285		
Fracture type (31A1 vs. 31A2)	1.9 (1.1–3.1)	**0.033**	1.6 (0.9–2.9)	0.102
Fracture side (left vs. right)	0.8 (0.6–1.2)	0.342		
Comorbidities				
None	1.0 (Reference)		1.0 (Reference)	
1	8.1 (1.9–34.0)	**0.004**	6.6 (1.6–28.2)	**0.010**
2 or more	16.9 (4.1–68.9)	** < 0.001**	8.65 (2.1–36.4)	**0.003**
ASA score (1 + 2 vs. 3 + 4)	6.8 (1.7–27.5)	**0.007**	5.1 (1.1–22.7)	**0.033**
Type of anesthesia				
General	1.0 (Reference)			
Combine spinal and epidural + spinal	0.8 (0.5–1.2)	0.192		
Nerve block	0.7 (0.2–3.1)	0.681		
Preoperative waiting time (per 1 day increase)	1.1 (1.1–1.2)	** < 0.001**	1.1 (1.0–1.2)	**0.005**
Hemoglobin (per 1 g/l increase)	1.0 (1.0–1.0)	**0.010**	1.0 (1.0–1.0)	0.907
Blood transfusion (no vs. yes)	2.2 (1.4–3.4)	**0.001**	1.7 (1.0–2.9)	0.052
CRQC grade				
Excellent	1.0 (Reference)		1.0 (Reference)	
Acceptable	2.2 (1.4–3.3)	**0.01**	1.6 (1.0–2.5)	**0.043**
Poor	5.9 (3.2–10.9)	** < 0.001**	6.7 (3.5–12.8)	** < 0.001**

*ASA* American Society of Anesthesiologists, *CRQC* Chang reduction quality criteria, *HR* hazard ratio, *CI* confidence interval

**Table 4:** Univariate and multivariate Cox regression analyses of factors associated with 1-year follow-up all-cause mortality

Variable	Univariate		Multivariate	
	HR (95% CI)	*P* value	HR (95% CI)	*P* value
Age (per 1 year increase)	1.1 (1.0–1.1)	**0.009**	1.0 (1.0–1.1)	0.066
Sex (Female vs. Male)	1.1 (0.6–1.8)	0.799		
Fracture type (31A1 vs. 31A2)	2.5 (1.1–5.7)	**0.036**	1.9 (0.8–4.6)	0.132
Fracture side (left vs. right)	0.8 (0.5–1.3)	0.275		
Comorbidities				
None	1.0 (Reference)			
1	21,624.4 (0–2.6E + 43)	0.828		
2 or more	55,076.5 (0–6.6E + 43)	0.812		
ASA score (1 + 2 vs. 3 + 4)	3.9 (1.0–15.8)	**0.060**	5.1 (1.1–23.8)	**0.040**
Type of anesthesia				
General	1.0 (Reference)			
Combine spinal and epidural + spinal	0.70 (0.4–1.2)	0.165		
Nerve block	0.8 (0.2–3.5)	0.815		
Preoperative waiting time (per 1 day increase)	1.1 (1.1–1.2)	**0.001**	1.1 (1.0–1.2)	**0.007**
Hemoglobin (per 1 g/l increase)	1.0 (1.0–1.0)	**0.001**	1.0 (1.0–1.0)	0.229
Blood transfusion (no vs. yes)	3.0 (1.7–5.6)	** < 0.001**	1.6 (0.8–3.2)	0.153
CRQC grade				
Excellent	1.0 (Reference)		1.0 (Reference)	
Acceptable	3.9 (2.1–3.3)	** < 0.001**	3.1 (1.6–5.9)	**0.001**
Poor	13.4 (6.4–7.4)	** < 0.001**	14.8 (6.8–32.1)	** < 0.001**

*ASA* American Society of Anesthesiologists, *CRQC* Chang reduction quality criteria, *HR* hazard ratio, *CI* confidence interval

The influencing factors screened by univariate Cox regression analysis were incorporated into a multivariate Cox proportional risk model. Increased age (HR 1.0, 95% CI 1.0–1.1), 1 comorbidity (HR 6.6, 95% CI 1.6–28.2), ≥ 2 comorbidities (HR 8.7, 95% CI 2.1–36.4), ASA scores 3 + 4 (HR 5.1, 95% CI 1.1–22.7), increased preoperative waiting time (HR 1.1, 95% CI 1.0–1.2), Acceptable CRQC grade (HR 1.6, 95% CI 1.1–2.5), and Poor CRQC grade (HR 6.7, 95% CI 3.5–12.8) were independent risk factors for all-cause mortality during the total follow-up (Table 3). ASA scores 3 + 4 (HR 5.1, 95% CI 1.1–23.8), increased preoperative waiting time (HR 1.1, 95% CI 1.0–1.2), and Acceptable (HR 3.1, 95% CI 1.6–5.9) and Poor CRQC grades (HR 14.8, 95% CI 6.8–32.1) were independent risk factors for all-cause mortality during the 1-year follow-up (Tables 4).

### Subgroup analyses

**Fig. 3:**
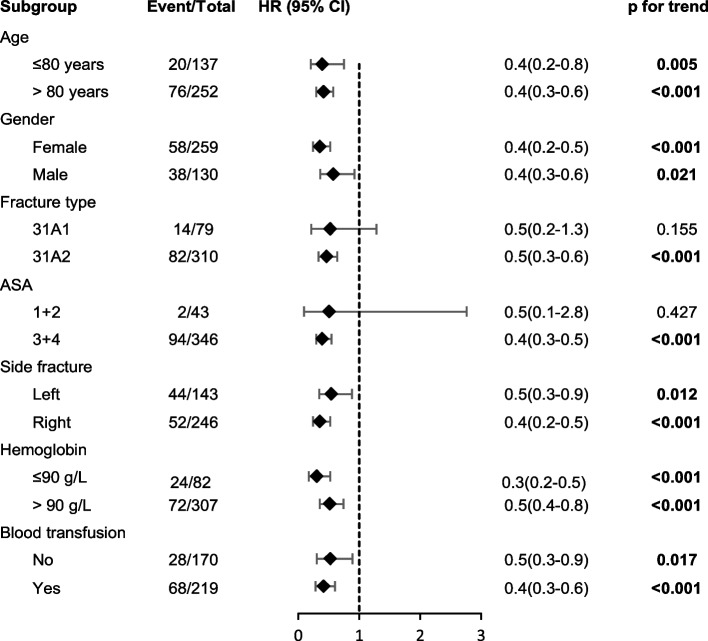
Forest plot of the subgroup analyses estimating the association between the Chang reduction quality criteria (CRQC) grade and total follow-up mortality based on different characteristics. Differences between subgroups were analyzed by Cox regression analysis. The black rhombuses represent the hazard ratio (HR), and the black horizontal lines represent the 95% confidence interval (CI)

**Fig. 4:**
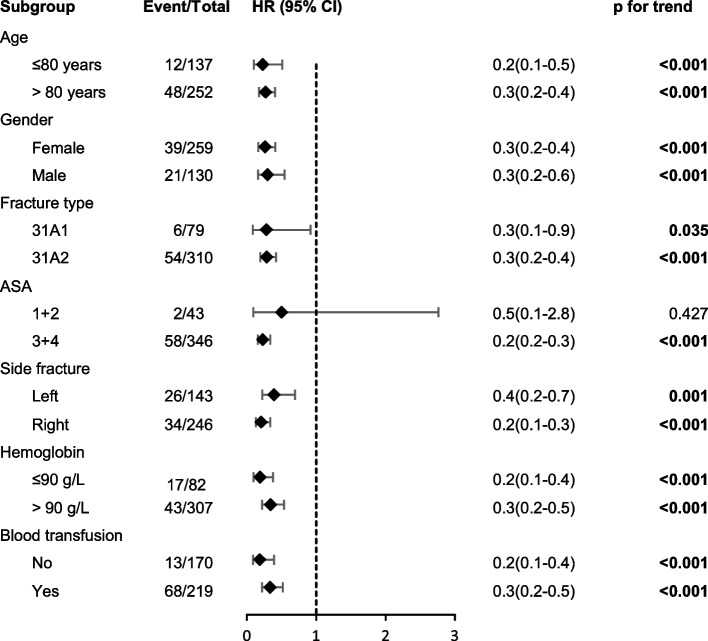
Forest plot of the subgroup analyses estimating the association between the Chang reduction quality criteria (CRQC) grade and 1-year follow-up mortality based on different characteristics. Differences between subgroups were analyzed by Cox regression analysis. The black rhombuses represent the hazard ratio (HR), and the black horizontal lines represent the 95% confidence interval (CI)

To further verify whether the predictive values of the CRQC for mortality were consistent across populations, we performed subgroup analyses using forest plots, as shown in Fig. 4. Consistent with the main analysis, subgroup analysis showed that in most subgroups, a lower CRQC grade was significantly associated with increased all-cause mortality (p for trend < 0.05). However, only two subgroups, patients with ASA scores 1 + 2 (*p* = 0.427) and patients with fracture type 31A2 (*p* = 0.155), were exceptions at the total follow-up. At the 1-year follow-up, only one subgroup, patients with ASA scores 1 + 2 (*p* = 0.427), was the exception (Figs. 3 and 4).

## Discussion

The key to stable fracture fixation is reduction quality. Many studies have included restoration quality, but only as a confounding variable, rather than as the main object of research [26–30]. This study focuses on the CRQC, which are a relatively new set of criteria for assessing the quality of reduction following fracture fixation. We verified that compared with patients with an Excellent CRQC grade, those with an Acceptable CRQC grade had a 0.6- and 1.1-times increase in mortality at the total postoperative and 1-year follow-up, respectively, while those with a Poor CRQC grade showed a 5.7- and 13.8-fold increase in mortality, respectively. In other words, fracture reduction quality appears to be an important predictor of postoperative mortality in older intertrochanteric fracture patients.

The potential factors of fracture reduction quality on mortality are pain and biomechanical changes. The main objective of intertrochanteric fracture surgery is early movement for reducing bed rest complications and mortality [8, 31]. However, studies have shown that poor reduction at the broken end of the fracture will make it very painful for the patient to move; thus, the patient will refuse to move, increasing the mortality rate [32]. In addition, instability at the broken end after poor fracture reduction can lead to limb shortening and biomechanical impairment, especially the loss of abductor function, which may also lead to the same outcome [33].

Currently, however, when the patient's risk of death is high, the surgeon accepts a poor reduction, as continuing the fixation and shortening the operation time are considered to be more important than attempting to further improve the reduction. This also explains why, in our study, a high number of comorbidities was associated with poor reduction quality. Similarly, the increased operative time, soft tissue dissection, and blood loss associated with open reduction relative to closed reduction may not be in the patient's best interest, or their functional needs may be lower, meaning they can better tolerate a poor reduction. The findings of this study urge us to better understand and focus more on the quality of the reduction.

At present, there are two main standards for evaluating the postoperative reduction quality of intertrochanteric fractures: the CRQC and the BRQC [16]. The greatest innovation of the CRQC lies in the introduction of the concepts of PMCS and NMCS [15, 34]. The CRQC require good reduction on anteroposterior (AP) views, meeting two conditions: 1. a displacement less than the bone cortex thickness; and 2. neutral or positive medial cortical support. The definition of good reduction thus excludes two possibilities: NMCS with displacement less than the cortical thickness or PMCS with displacement greater than the cortical thickness of the fracture. The CRQC require good reduction on lateral views and a smooth anterior cortex, meaning that the displacement must be less than half of the bone cortex thickness, emphasizing the strong support of the anterior cortex [35, 36]. Currently, most internal fixation materials do not immobilize small trochanter fragments, so the CRQC do not explicitly require posterior cortical alignment [29]. The CRQC are also more reliable than the BRQC for three main reasons. First, the use of the BRQC may result in the loss of some details. For example, in the BRQC, nonalignment includes three possible conditions: poor alignment only on the AP surface; poor alignment only on the side; and poor AP and lateral alignment; the BRQC cannot distinguish between these conditions. In contrast, the CRQC uses a more ideal, 4-point scoring system and retains more details [16]. Second, the CRQC reasonably adopt the concepts of PMCS and NMCS. PMCS provides cortical support between the two main fragments and prevents further lateral sliding of the femoral head and neck fragments; NMCS, on the other hand, is defined as contact between the proximal bone cortex and the distal cancellous bone and embedding of the proximal bone mass in the low-density rotor region, promoting further lateral sliding of the femoral head and neck fragments, which in turn leads to internal fixation failure [15]. In the BRQC, a good AP view reduction means a displacement of less than 4 mm, but PMCS and NMCS can both occur under the premise of a displacement of less than 4 mm, and ultimately, PMCS and NMCS can lead to different clinical outcomes [16]. However, the CRQC can be a good solution to this problem. Third, using one or half cortical thickness to describe the displacement is better than using the actual distance of 4 mm because it can be measured directly with the naked eye through intraoperative C-arm fluoroscopy without the use of special tools.

Many previous studies have confirmed that increased age and number of comorbidities were independent risk factors for hip fracture survival in older adults [17, 18]. Cui et al. [37], for example, found that mortality was positively correlated with age. Lei et al. retrospectively analyzed 1057 hip fracture patients aged above 60 years and found that comorbidities were correlated with 5-year mortality after surgery [38]. Our study found that mortality increased with age and the number of comorbidities during the total follow-up. The reason is that more diseases or advanced age will accelerate the degeneration of physiological reserve function of multiple organ systems, the body will continue to be in a state of consumption, and the ability of organs to resist surgery and anesthesia will be gradually weakened, decreasing the postoperative survival rate [39]. However, at the 1-year follow-up, there were no corresponding conclusions. We speculate that the small sample size and short follow-up period may have contributed to some of the differences in the studies.

Some studies have reported that mortality is associated with higher ASA scores [18, 40]. Capkin et al. [41] suggested that an ASA score of ≥ 3 was associated with mortality. The ASA score is used to assess the "disease state" or "physical status" of the patient before surgery: the higher the ASA score is, the more severe the disease and the higher the mortality. Similarly, our study further demonstrated that patients with ASA scores of 3 + 4 had a higher risk of death at the total- and 1-year follow-ups than patients with ASA scores of 1 + 2.

The role of preoperative waiting time as a factor associated with the risk of death remains controversial. Chang [42] conducted a meta-analysis and found that delaying the operation time significantly increased mortality, similar to the findings of our study. However, other studies have shown that early surgery does not improve the prognosis of older adult patients with hip fractures [43, 44]. We think that making full use of the "window period" when the underlying disease has not been aggravated after fracture, completing early surgery and helping patients stand as soon after the operation as possible to facilitate basic disease control, prevent fracture hypostatic pneumonia caused by lying in bed, bedsores, urinary tract infections, and deep venous thrombosis complications and reduce mortality.

However, this study had some limitations. This was a retrospective single-center study; large-sample, multicenter, randomized controlled studies are needed to confirm the results. Second, we only evaluated medium-term outcomes because the number of deaths was relatively small, and it was impossible to assess short-term outcomes.

## Conclusion

In conclusion, we demonstrate that postoperative mortality in older intertrochanteric fracture patients is associated with reduced reduction quality according to the CRQC. Therefore, we strongly recommend that orthopedic surgeons consider reduction quality when treating older intertrochanteric fracture patients to improve their outcomes.

## Data Availability

The datasets generated and/or analyzed during the current study are not publicly available, as they contain information that could compromise the privacy of research participants, but are available from the corresponding author on reasonable request.

## Abbreviations

CRQC: Chang reduction quality criteria
ASA: American Society of Anesthesiologists
HR: Hazard ratio
CI: Confidence interval
